# Multifaceted Role of Probiotics in Enhancing Health and Growth of Aquatic Animals: Mechanisms, Benefits, and Applications in Sustainable Aquaculture—A Review and Bibliometric Analysis

**DOI:** 10.1155/anu/5746972

**Published:** 2025-02-22

**Authors:** Madhulika, Soibam Ngasotter, Maibam Malemngamba Meitei, Tao Kara, Martina Meinam, Sanjeev Sharma, Sanjaykumar Karsanbhai Rathod, Sanjenbam Bidyasagar Singh, Soibam Khogen Singh, Raja Aadil Hussain Bhat

**Affiliations:** ^1^ICAR-Central Institute of Fisheries Education, Mumbai 400061, India; ^2^College of Fisheries, Central Agricultural University, Agartala 799210, Tripura, India; ^3^College of Fisheries Science, CCS Haryana Agricultural University, Hisar 125004, Haryana, India; ^4^Krishi Vigyan Kendra, ICAR—Research Complex for NEH Region, Manipur Centre, Ukhrul 795142, Manipur, India; ^5^ICAR-Directorate of Coldwater Fisheries Research, Bhimtal 263136, India

**Keywords:** aquaculture, bibliometrics, gut health, nutrition, probiotics

## Abstract

Probiotics play a pivotal role in enhancing the health and growth of aquatic animals in aquaculture. These beneficial microorganisms contribute to improved digestion and nutrient absorption by producing digestive enzymes such as amylases, proteases, and lipases, besides providing essential nutrients. By creating a favorable microbial balance in the gastrointestinal tract (GIT), probiotics reduce harmful microorganisms and promote the proliferation of beneficial bacteria like *Lactobacillus* spp. and *Bifidobacterium* spp. This modification of gut microflora leads to more efficient digestion and significantly enhances overall health and growth performance in fish. Additionally, probiotics produce antimicrobial substances, such as bacteriocins and organic acids, which inhibit pathogenic bacteria and bolster disease resistance. They also play a crucial role in improving water quality in aquaculture systems by aiding in the turnover of organic nutrients and reducing toxic substances. Incorporating probiotics into aquaculture practices has demonstrated considerable potential in boosting the productivity and health of aquatic animals, making them an essential component of sustainable aquaculture. This review delves into the multifaceted benefits of probiotics, including enhanced feed utilization, immune responses, and pathogen resistance, and elucidates the mechanisms underlying these effects. Furthermore, it includes a bibliometric analysis of the past 30 years, providing a comprehensive overview of research trends and advancements in this field.

## 1. Introduction

The aquaculture industry is rapidly expanding day by day to meet the nutritional demands of the world population. As a crucial global industry, aquaculture faces the challenge of sustainable growth and resource optimization. As the fastest-growing food production method, aquaculture holds significant promise for addressing food insufficiency and malnutrition while meeting the world's rising food demands [1]. Governments and development agencies have increasingly recognized the potential of fish farming, given its critical role in sustaining global food security. Despite its potential, aquaculture faces several significant challenges, including disease outbreaks, environmental impacts, and the need for sustainable feed resources [2]. While proper husbandry practices can reduce stress in aquaculture systems, it is impossible to eliminate stress entirely, which ultimately leads to increased disease susceptibility in farmed fish [3]. Traditionally, chemicals and antibiotics have been used to manage disease outbreaks in aquaculture alongside vaccines and biocontrol agents. However, the use of these treatments has been banned or restricted in many countries due to their potential risks to consumers and the environment, such as bioaccumulation and residues in fish tissues [3–5]. Additionally, the widespread use of antibiotics raises concerns about the development of antibiotic-resistant bacterial strains [4].

One promising approach to addressing these challenges is the use of probiotics—beneficial microorganisms that enhance the health and productivity of cultured species [6]. Probiotics are live microorganisms that, when administered in adequate amounts, confer health benefits to the host [7]. In aquaculture, probiotics are primarily used to improve growth performance, feed efficiency, and disease resistance. They achieve this by modulating the gut microbiota, enhancing innate immune responses, and imparting several other benefits [2]. The gut microbiota plays a crucial role in the overall health of aquatic animals, affecting various physiological processes such as digestion, nutrient absorption, and immune function [8, 9]. The dynamic interaction between the host and its gut microbiota is essential for maintaining health and preventing disease. Probiotics can also play a significant role in enhancing rearing water quality in aquaculture systems [3]. By promoting the decomposition of organic matter and reducing harmful substances like ammonia and nitrite, these beneficial microorganisms help maintain a healthier aquatic environment [10]. Improved water quality not only fosters better growth conditions for aquatic organisms but also reduces the incidence of disease outbreaks, contributing to more sustainable and productive aquaculture practices [11]. The increasing demand for environmentally sustainable methods in aquaculture has led to an increase in research on the application of probiotics in aquaculture. Adding probiotics to the diets of aquatic animals can improve growth, feed utilization, and physiological parameters [5, 7]. Probiotic dietary intake can enhance growth [12, 13], increase cell proliferation to support the immune response, especially in times of stress [14, 15]. In certain situations, additional probiotics may be needed to stimulate the immune system even further [16].

The recent trends in probiotics within aquaculture highlight significant growth and advancements in research, publications, and the involvement of various organizations and countries. These trends can be systematically analyzed by reviewing the published literature in this field, utilizing tools like scientometrics or bibliometric analysis. Bibliometric analysis involves the quantitative study of science to evaluate developments in science, technology, and innovation [17]. This approach assesses research publications using two main methods: performance analysis and science mapping [18]. Bibliometric analysis offers several advantages, such as uncovering emerging trends in article and journal performance, revealing collaboration patterns, identifying key research constituents, enhancing research positioning, and expanding research territories [18].

This review explores probiotics, focusing on their sources, modes of action, health benefits, and potential applications in fish nutrition, production, and health management. Furthermore, a bibliometric analysis is employed to identify research gaps, analyze publication trends and relationships, and evaluate co-word occurrences. By advancing the understanding and utilization of probiotics, this review aims to address critical challenges in the aquaculture industry, promoting sustainable and efficient practices.

## 2. Methodology

### 2.1. Literature Search

The literature search for this review was conducted using Google Scholar with key terms such as “probiotics in aquaculture,” “fish health,” “growth performance,” “mechanisms of action,” and “sustainable aquaculture,” applying Boolean operators (e.g., AND, OR) to refine results. Inclusion criteria focused on peer-reviewed, English-language publications, including review articles, original research papers, short communications, conference proceedings, and book chapters, while exclusion criteria filtered out irrelevant, non-English, or duplicate studies.

### 2.2. Bibliometric Analysis

For bibliometric analysis, we collected data from 1995 to 2025 using Scopus, a comprehensive, multidisciplinary, and trusted abstract and citation database of peer-reviewed literature, including scientific journals, books, and conference proceedings. The data search was conducted using the keywords “Probiotics” AND “Aquaculture” OR “Fish” OR “Shellfish” in the “Article titles, Abstracts, and Keywords.” We then filtered the data to the subject areas “Agriculture and Biological Sciences,” “Immunology and Microbiology,” “Environmental Science,” “Pharmacology, Toxicology and Pharmaceutics,” “Veterinary,” “Biochemistry, Genetics and Molecular Biology,” and “Medicine.” Data for the year 2025 were excluded as it is an upcoming year. Consequently, we exported 4,855 bibliographic records.

## 3. Role of Gut Microbes

The microbes in the gastrointestinal tract (GIT) exist in a natural balance known as “symbiosis,” similar to other biological systems. This balance includes “symbionts,” which benefit from a mutualistic relationship with the host; “commensals,” which neither help nor harm the host; and certain potentially harmful microorganisms [19]. It is well recognized that the microbiota of healthy fish offers several health benefits, including metabolism, immune regulation, nutrition, and pathogen defence [2, 5]. The gut microbiota, consisting of microorganisms living in the intestinal tract, plays a crucial role in various biochemical processes and influences the host's immune system [5]. This microbial community is dynamic and constantly changing. Microbes in the gut can be classified as transient or persistent based on their duration of stay. Transient microbiota enters the gut through diet and remains for a short period before being displaced by resident microbes that adhere to the gut wall [9]. In contrast, resident microbiota establishes a lasting symbiotic relationship with the host, persisting within the intestinal membrane. When this balance is disrupted, resulting in changes in bacterial composition, diversity, or function—such as a decrease in symbiotic bacteria or an increase in pathogenic microorganisms—a condition called “dysbiosis” occurs [7]. Dysbiosis undermines the protective function of the gut microbiota, potentially leading to various illnesses, decreased performance, and discomfort [20].

The gut microbiota significantly impacts several aspects of fish biology, including physiology, development, lifespan, immunity, and pathogen defence, making it essential for fish fitness [13]. Recent reviews have explored bacterial community diversity and functions in healthy fish, as well as external factors affecting fish gut microbiota and the interactions between gut microbiota and innate immunity in fish [2–5, 20].

## 4. Probiotics

The term “probiotics” originates from a Greek word meaning “for life” and refers to living nonpathogenic organisms and their beneficial effects on hosts [3]. The concept of “probiotics” was first introduced by Vergin, who observed the positive effects of “probiotika” on gut microflora while studying the harmful impacts of antibiotics and other microbial substances [5]. Lilly and Stillwell [21] later redefined probiotics as a product produced by one microorganism that stimulates the growth of another microorganism. Fuller [22] further refined the definition to “non-pathogenic microorganisms which, when ingested, exert a positive influence on the host's health or physiology.” The most recent definition, proposed jointly by the Food and Drug Administration (FDA) and World Health Organization (WHO), describes probiotics as “live microorganisms which, when administered in adequate amounts, confer a health benefit to the host” [23].

Probiotics interact with both the host and microbiota, delivering benefits through their immunogenic potential, production of antimicrobial molecules, enhancement of mucosal integrity, and competition with pathogens for adhesion sites and nutrients [11] By producing chemicals like bacteriocins and organic acids and by competitive exclusion, they can boost the population of advantageous microorganisms like *Lactobacillus* spp. and *Bifidobacterium* spp., which suppress harmful bacteria [7]. The effectiveness of probiotics is closely related to the specific microbial strain, leading to the use of different multistrain combinations to achieve synergistic effects [3]. Probiotics are known for numerous benefits, one of the most significant being the modulation of the immune system. Probiotics have also been identified as effective immunomodulatory agents in shellfish, such as shrimp [24]. The role of probiotics in immune modulation has been extensively researched and reviewed in both humans and animals. Numerous immunological studies have been conducted on various fish species using different probiotics, demonstrating their remarkable ability to stimulate fish immunity in both *in vivo* and *in vitro* conditions [3, 14].

The range of probiotics used in aquaculture includes Gram-negative and Gram-positive bacteria, yeasts, bacteriophages, and unicellular algae [8, 25]. Microorganisms selected as probiotics must be nonpathogenic to animals and able to survive in the low pH and high bile concentration of the GIT. Most probiotics come from the *Lactobacillus* and *Bifidobacterium* genera due to their recognized safety and common presence in the GIT [26]. The use of spore-forming bacteria as probiotics is growing, especially from the *Bacillus* genus [27]. *Bacillus* spp. spores can resist various physical and environmental factors, including heat, desiccation, and UV radiation, allowing them to remain viable during feed pelleting, storage, and handling [6].

## 5. Probiotics in Aquaculture

Numerous approaches have been investigated to modulate gut microbiota in order to enhance feed utilization and health status, which is crucial for food digestion quality [8].  Table 1 represents the effects and applications of probiotics in aquaculture.

The microbiome is defined as the microbial communities inhabiting specific environments, encompassing the microorganisms and their activities [20]. The microbiota includes bacteria, protozoa, fungi, and algae, while “their theater of activity” involves microbial metabolites, structures, genetic elements (like phages and viruses), and relic DNA embedded in the habitat [2, 20]. Probiotics have garnered significant interest as functional feed additives in various aquaculture species [8]. Recently, a wide range of bacteria and yeast have been used as probiotics in aquaculture, including *Lactobacillus* spp., *Lactococcus* spp., *Leuconostoc* spp., *Enterococcus* spp., *Carnobacterium* spp., *Shewanella* spp., *Bacillus* spp., *Aeromonas* spp., *Vibrio* spp., *Enterobacter* spp., *Pseudomonas* spp., *Clostridium* spp., and *Saccharomyces* spp. [2, 20]. These microorganisms are commonly found in the digestive tracts of host animals [14].

**Figure 1:**
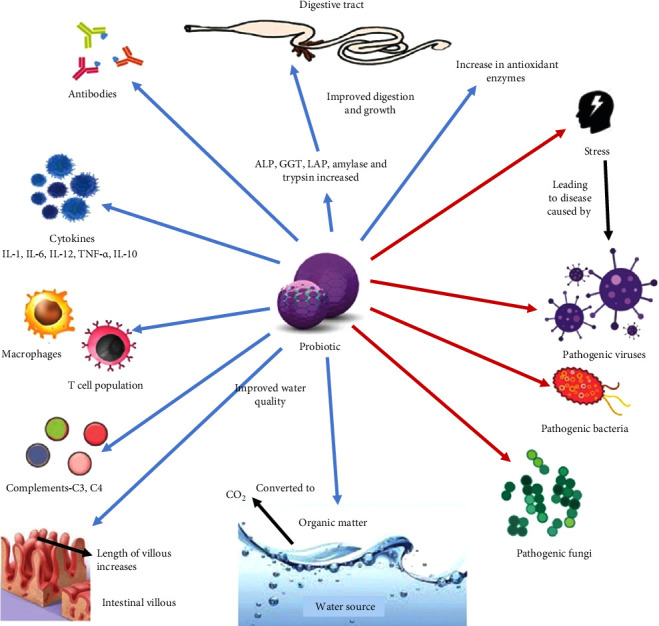
Beneficial effects of probiotics in aquaculture. Blue arrows indicate a positive effect. Red arrow indicates inhibitory effect. ALP, alkaline phosphatase; GGT, gamma glutamyl transferase; LAP, leucine aminopeptidase; IL, interleukin. Reprinted from Vijayaram and Kannan [37] under creative commons license https://creativecommons.org/licenses/by-nc-sa/3.0/deed.en.

In aquaculture, probiotics fulfill multiple roles in benefiting host animals. They demonstrate antiviral properties against infections and produce inhibitory compounds [19]. Probiotics also contribute to better water quality by recycling organic nutrients and decreasing harmful substances ammonia in aquaculture systems [11, 36]. They enhance immune responses by boosting the phagocytic activity of leukocytes [19], and compete for nutrients that pathogenic microbes would otherwise utilize. Additionally, probiotics compete for adhesion sites and food with pathogens on the gut epithelial surface, preventing their colonization [25]. Furthermore, they act as nutrient sources while secreting various enzymes such as amylase, lipase and protease; and enhance feed degradation and assimilation, thereby improving their nutritional value [36]. Some beneficial effects of probiotics in aquaculture are shown in Figure 1.

## 6. Mode of Action

The activities of bacterial probiotics are multifaceted, involving the regulation of host animal immune responses as well as interactions with gut bacterial ecosystems [14]. Probiotics work through various mechanisms that are not yet understood completely, likely acting either in the gastrointestinal (GI) lumen or on the GI tract wall [19]. Given the continuous interaction between the intestinal microbiota in aquatic animals and the environment and host functions, probiotics are defined as live microbial adjuncts that confer beneficial effects by: (i) modifying the host-associated or ambient microbial community [28, 30], (ii) improving feed utilization or enhancing its nutritional value [33], (iii) boosting the host's response to diseases [29–32], and (iv) improving the quality of the ambient environment [28, 35].

Common effects of probiotic supplementation have been reported, including improved feed utilization and increased weight in fish and shellfish [38, 39]. Furthermore, by breaking down indigestible components, generating vitamins, and detoxifying food chemicals, probiotics can improve feed palatability and boost host hunger [40]. According to Yan, Boyd, and Burgess [41], they produce compounds like peroxide, bacteriocins, siderophores, and lysozyme enzymes, and they also exert physiological and immunological effects. They use these strategies to prevent pathogens by competing for attachment sites [42] and by manufacturing other chemicals. Probiotics also have the ability to modify the gut microbiota by inhibiting pathogenic bacteria and bolstering the host's defences by producing chemicals known to be inhibitory [40]. Certain probiotics adhere to the intestinal mucosal layer, blocking pathways commonly used by pathogenic bacteria [5]. They also increase the resistance of aquatic animals to environmental stressors during aquaculture activities [2, 4]. The mechanisms mentioned provide initial insights into the beneficial effects of probiotics in aquatic animals. However, further research involving transcriptomic and proteomic analyses is needed to deepen the understanding of probiotic activity and host interactions.

## 7. Gut Microbiota Modulation

A key factor in maintaining a healthy GIT is the composition of its microbial population. Probiotics can alter the dynamics of the microbial population in the GIT, fostering a more favorable microbial environment by shifting the balance towards beneficial microbes over harmful ones [20, 26]. Healthy gut microbiota is frequently associated with better immunity, more effective digestion, and increased animal performance [14, 19]. The decrease in pathogenic microbes within the GIT can be ascribed to the synthesis of antimicrobial agents, such as bacteriocins [43], as well as the attachment of probiotic microbes to the intestinal epithelium. This process serves to either drive out pathogens by means of competition or by inducing an immune response [6].

Probiotics typically cause populations of *Lactobacillus* spp. and *Bifidobacterium* spp. to rise, while coliform populations fall in the GIT microflora [11]. Every popular probiotic bacterium, including lactic acid bacteria (LAB), exhibits this change in flora [2]. *Lactobacillus* spp. and *Bifidobacterium* spp. are two examples of good microorganisms whose populations can be increased by probiotics. These microorganisms then prevent the growth of harmful microbes by creating chemicals like bacteriocins and organic acids, as well as by competitive exclusion [24].

## 8. Nutrient Utilization

The amount and activity of endogenous enzymes in fish are insufficient for the complete metabolism of ingested food. Therefore, enzymes produced by permanent gut endosymbionts and probiotics are vital from a nutritional perspective [3]. Previously, there has been extensive investigation into the production of extracellular enzymes by various fish probiotic strains [32, 35, 44, 45]. They also provide essential nutrients like vitamins, fatty acids, and amino acids, contributing to the digestive processes and feed utilization, which in turn improves the health, growth performance, and well-being of aquatic species [4, 44–46]. Studies have examined changes in enzyme patterns due to probiotic intake in aquaculture species, with notable increases in the secretion of enzymes like amylase, trypsin, protease, and lipase observed in sea bass (*Dicentrarchus labrax*) [47] and rohu (*Labeo rohita*) [48] fed with a live probiotic mixture. These enhancements in nutrient digestibility and overall health are likely due to the increased availability of nutrients produced by probiotics in aquafeeds [44, 46].

Numerous fish species fed diets containing beneficial bacterial strains such as *B. cereus*, *B. subtilis*, *B. licheniformis*, and *E. faecium* have demonstrated enhanced feed utilization and growth performance [13, 28, 32, 33, 49]. The inclusion of *Bacillus* spp. in fish diets has notably improved digestive activities due to their capability to produce exoenzymes [11]. Similarly, white shrimp species *L. vannamei* and *F. indicus* have shown improved digestion of dry matter, crude protein, and phosphorus with *Bacillus* spp. supplemented diet [35, 38]. Furthermore, spore-forming bacteria such as *Bacillus amyloliquefaciens*, *Bacillus aerius*, and *Bacillus sonorensis* produce extracellular enzymes, including *α*-amylase, cellulase, proteases, and metalloproteases, which enhance nutrient digestion [50, 51]. Increased enzyme activity in the GIT of animals supplemented with probiotics may result from the enzymes produced by the probiotics themselves or from changes induced in the microbial population that, in turn, affect enzyme production [3].

## 9. Growth Promotion

Several studies have demonstrated that probiotics positively impact growth performance in aquaculture. Probiotics function as sources of nutrients, vitamins, and digestive enzymes, enhancing feed utilization, nutrient absorption, and overall growth performance [2, 4, 5, 8]. One key benefit of probiotics is their ability to improve gut health. By colonizing the GIT, probiotics create a balanced microbial environment, reducing the presence of harmful pathogens. This balance promotes better digestion and nutrient absorption, directly contributing to enhanced growth [44]. For instance, studies have shown that probiotics improve the digestibility and appetite of aquatic species, leading to higher survival rates and improved growth. They improve intestinal microbes' appetite, digestibility, and balance, leading to enhanced survival and growth in aquatic species [14, 44].

Additionally, probiotics have been used in phytoplankton, the foundation of aquatic food chains, to enhance nutrient production [33, 52]. Moreover, probiotics have been particularly effective in low-protein diets, making them valuable in cost-effective aquaculture practices [36]. By improving the digestibility, efficiency, and feed conversion ratios (FCR) in aquaculture species, they help reduce production costs without compromising growth performance [3]. In aquaculture, many studies have been done to see the effects of probiotics on growth, such as effects of *Lactococcus lactis* on enhanced growth performance in *L. vannamei* [15], *E. casseliflavus* (EC-001) on growth performance of common carp [16], *B. subtilis* on the growth performance of *L. vannamei* [32], *Bacillus* spp. on growth in the Indian white shrimp *F. indicus* [35], *L. acidophilus* on the growth performance in African Catfish (*Clarias gariepinus*) [39].

## 10. Immunity, Disease Resistance, and Antioxidant Activity

In fish aquaculture, probiotics have been shown to significantly enhance antioxidant status, immunity, and resistance to pathogens. Probiotics increase the activity of antioxidant enzymes such as superoxide dismutase, catalase, and glutathione peroxidase, which reduce oxidative stress and improve the overall health of fish [3, 4]. These beneficial microbes also bolster the immune system by stimulating both innate and adaptive immune responses [6]. They enhance the phagocytic activity of leukocytes, increase the production of immunoglobulins, and modulate cytokine production, thereby strengthening the fish's immune defence [2]. Furthermore, probiotics improve fish resistance to pathogens through several mechanisms, including competitive exclusion, where probiotics outcompete harmful microbes for adhesion sites and nutrients in the gut [24]. Probiotics also produce antimicrobial substances like bacteriocins and organic acids that inhibit pathogen growth [5, 8]. By creating a more balanced and beneficial gut microbiota, probiotics help prevent infections and diseases, leading to better survival rates, improved growth, and overall enhanced productivity in fish aquaculture [32].

Studies have demonstrated that fish-fed probiotics at concentrations ranging from 10^5^ to 10^9^ cells for 15 days exhibited increased resistance to pathogens. However, fish-fed probiotics at the same concentration for 8 weeks did not show a higher survival rate compared to control groups [53]. Probiotics have the ability to produce useful molecules like siderophores, bacteriocins, antibiotics, enzymes (such as lysozymes and proteases), and hydrogen peroxide, in addition to changing the pH of the intestine by producing organic acids [20]. The inclusion of probiotics in aquafeeds has been said to inhibit intestinal diseases in several cultured species [54–56]. Notable species like Japanese flounder (*P. olivaceus*) [31], Atlantic salmon (*Salmo salar* L.) [57], and rainbow trout (*Oncorhynchus mykiss*) [57] have shown increased resistance to infections when fed probiotic-supplemented diets. These fish exhibited enhanced immune responses, including improved neutrophil migration, lysozyme activity, and bactericidal activities, resulting in greater resistance to bacterial pathogens such as *Edwardsiella* spp., *Aeromonas* spp., *Flavobacterium* spp., *Photobacterium* spp., and *Vibrio* spp. [31, 57].

## 11. Water Quality

Probiotics play a vital role in enhancing water quality in aquaculture systems. They promote the decomposition of organic matter and reduce harmful substances like ammonia (NH_3_) and nitrite (NO_2_^−^), thereby maintaining a healthier aquatic environment [11, 36]. These beneficial microorganisms facilitate the turnover of organic nutrients, improving water clarity and minimizing the risk of toxic buildup [3]. Probiotics also help balance microbial populations in the water, outcompeting pathogenic bacteria and preventing their proliferation [11].

According to a number of studies, probiotics, including bacteria and yeast, improve fish habitats by limiting the growth of dangerous phytoplankton and pathogenic bacteria, as well as by bioremediating organic wastes and pollutants in aquaculture waterbodies [3, 34]. For fish, prawns, and mollusk farming operations, probiotics are produced commercially in a variety of specialized preparations [58]. By removing harmful elements from the water, living probiotic cells in the waterbodies enhance water quality and aquatic animal health [10]. As a result, probiotics for aquaculture are offered in a variety of forms, such as water additives and feed supplements. These improvements create better growth conditions for aquatic organisms and reduce disease outbreaks, contributing to more sustainable and productive aquaculture practices.

## 12. Bibliometric Analysis

The bibliometric analysis identifies research gaps, evaluates publication trends and relationships, and investigates co-word occurrences. This section provides a detailed examination of publication trends related to probiotics in aquaculture, explores patterns of co-authorship across countries, and analyzes the co-occurrence of key terms within this field.

### 12.1. Publication Trends, Mode of Documentation, and Source of Publication

**Figure 2:**
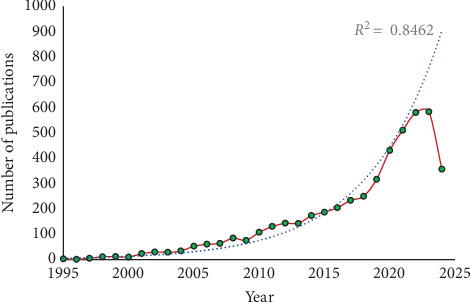
Growth trends in the publications of probiotics in aquaculture.

According to Scopus data, there were only three publications on probiotics in aquaculture indexed in 1995 and has grown to 4,855 publications by 2024. The growth pattern, illustrated in Figure 2, shows an exponential trend line with an R^2^ value of 0.8462, indicating a consistent growth rate in publications on probiotics in aquaculture from 1995 to 2024. However, there is a slight decrease in publications in 2024, with a total of 357, due to it being the middle of the current year (data collection done in July 2024).

**Figure 3:**
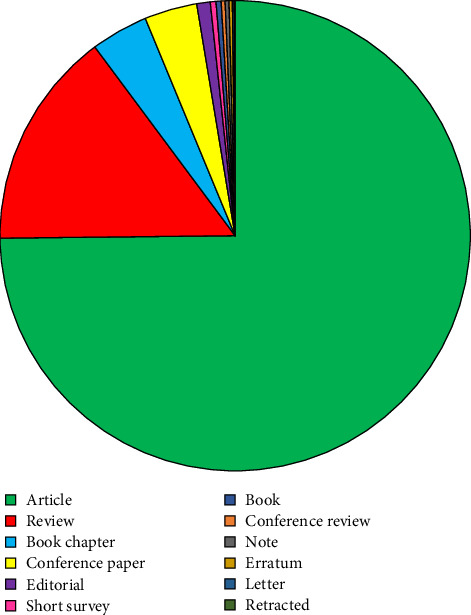
Pie chart showing the different forms of publication of probiotics in aquaculture.

Most of these publications are research articles, numbering 3634 (75%), followed by review articles (728), book chapters (191), conference papers (176), editorial articles (45), short surveys (19), books (17), conference reviews (13), notes (13), errata (11), and letters (6). There were also three retracted papers.  Figure 3 presents a pie chart showing the distribution of different publication types on probiotics in aquaculture.

The majority of publications on probiotics in aquaculture appeared in the journal “Aquaculture,” published by Elsevier, with 345 articles (7.1% of the total). This is followed by “Fish and Shellfish Immunology,” also by Elsevier, with 331 articles (6.8%), “Aquaculture Research” by Wiley, with 174 articles (3.6%), “IOP Conference Series: Earth and Environmental Science” with 112 articles (2.3%), and “Aquaculture International” by Springer Nature with 111 articles (2.3%).

### 12.2. Subject-Wise Distribution

The domain encompasses multidisciplinary subjects, with a total of 4855 papers distributed across various fields. To streamline our analysis, we have focused on specific areas of interest, which has also reduced the number of publications and simplified the analysis. This domain covers various aspects, including immunological aspects, gene extraction and analysis of probiotics, and their pharmaceutical potential. However, most publications on probiotics in aquaculture are related to agricultural and biological sciences, followed by immunology, microbiology, and environmental sciences.

### 12.3. Most Prolific Authors and Countries

The most prolific authors have been ranked based on their total number of publications.  Table 2 lists the top 10 authors and countries with the highest number of publications in the field of probiotics in aquaculture. China leads in publications with 948, followed by India with 578 and the USA with 506. Notably, the three papers from 1995 were published by researchers from Belgium, Scotland, and Ireland, focusing on experimental work with turbot fish.

### 12.4. Co-Authorships by Countries

Co-authorship networks illustrate the collaborative relationships and idea-sharing among authors from various organizations or countries. Analyzing these networks can reveal differences in research organization specific to a field [59] and the impact of geographical distance on collaboration [60, 61]. Visualization techniques, such as VOSviewer, have been employed to represent these network structures and their properties. VOSviewer, a novel mapping technique that serves as an alternative to the well-known multidimensional scaling (MDS) [62], is used in this study to map co-authorship networks. VOSviewer aims to position items in a low-dimensional space such that the distance between any two items accurately reflects their similarity or relatedness [62].

**Figure 4:**
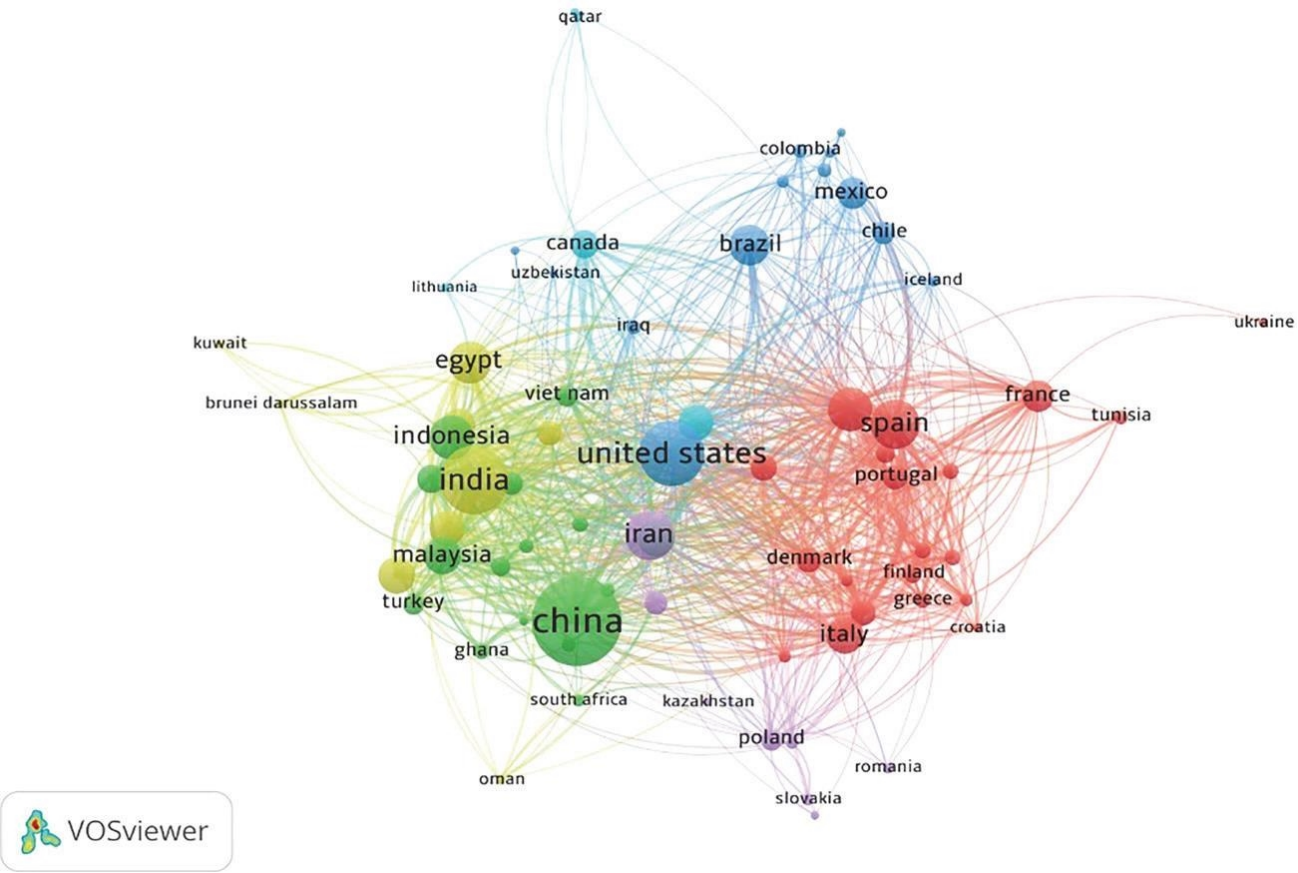
The co-authorships network of the countries. The node circle represents the countries, the size of the node circles represents the number of publications and the links represent co-author links, and the thickness of the links represents the number of co-authors.

The co-authorship network of the countries is shown in Figure 4. To analyze co-authorship among countries in the context of probiotics in aquaculture, a maximum of 25 countries per document and a minimum of 5 documents per country were selected. Out of 149 countries, 71 met the threshold, and the total strength of the co-authorship links among these 71 countries was calculated (as shown in Figure 4). The clustering technique in VOSviewer [63] for mapping co-authorship networks consists of clusters representing groups of collaborating countries or researchers, revealing patterns of scientific collaboration [59]. Clusters located close to each other indicate closely related collaborations [63]. The size of the nodes indicates the countries with the highest number of publications, while the size of the connecting links indicates the strength of collaboration between countries.

In the current analysis, the network comprises six clusters. Cluster I (red) is the largest, consisting of 20 items: Austria, Belgium, Croatia, Denmark, Finland, France, Germany, Greece, Hong Kong, Ireland, Israel, Italy, Netherlands, Portugal, Spain, Sweden, Switzerland, Tunisia, Ukraine, and the United Kingdom. Cluster II (green) includes 16 items: Australia, Bangladesh, China, Ghana, Indonesia, Malaysia, New Zealand, Nigeria, Pakistan, Philippines, Singapore, South Africa, Taiwan, Turkey, United Arab Emirates, and Vietnam. Cluster III (purple) consists of 13 items: Argentina, Brazil, Chile, Colombia, Cuba, Ecuador, Iceland, Iraq, Mexico, Peru, Sri Lanka, United States, and Uzbekistan. Cluster IV (yellow) comprises nine items: Brunei Darussalam, Egypt, India, Japan, Kuwait, Oman, Saudi Arabia, South Korea, and Thailand. Cluster V (violet) includes eight items: Czech Republic, Iran, Kazakhstan, Poland, Romania, Russian Federation, Serbia, and Slovakia. Cluster VI (blue) consists of five items: Canada, Lebanon, Lithuania, Norway, and Qatar.

### 12.5. Co-Occurrence of Keywords

Co-occurrence analysis of keywords is a powerful technique for mapping scientific fields and tracking their evolution. This method has been used across various domains, including bibliometrics [64], informetrics [65], and complexity sciences [66]. Researchers derive keywords from sources such as titles, abstracts, and citation contexts [67]. This analysis identifies frequently used terms, central concepts, and the relationships between topics within a field. Co-word occurrence maps can illustrate shifts in research focus and maturity over time. For instance, in complexity sciences, there has been a notable transition from general, foundational concepts to more applied and specific keywords [66].

Co-occurrence analysis visually represents bibliometric networks of keywords, highlighting emerging research areas and evolving themes. In this study, VOSviewer was utilized to create a co-occurrence network of keywords related to probiotics in aquaculture, displaying it on a two-dimensional map. Keywords co-occur when they appear together in the same title, abstract, or citation context [66]. Each node represents a keyword, with its size reflecting the total number of occurrences [17]. The distance between nodes indicates the similarity or relatedness of keywords, with more frequently co-occurring terms positioned closer together. VOSviewer uses clustering techniques to group keywords based on their co-occurrence, with clusters that are nearer on the map indicating closer relationships [60].

**Figure 5:**
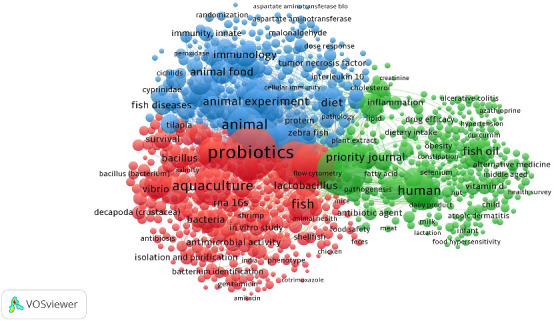
Network visualization of the co-occurrence of keywords. Each node represents the number of keywords.

In this study, a minimum co-occurrence threshold of 10 was set, resulting in 2,053 keywords meeting the criteria out of 23,619 total keywords, with 1,000 keywords selected by default. Frequently occurring keywords with the highest links include “probiotics,” “probiotic agents,” “aquaculture,” and “non-human animals.” Three clusters emerged from the analysis: Cluster I (red) with 380 items (e.g., probiotics, RNA 16S, aquaculture, bacteria, shrimps), Cluster II (green) with 346 items (e.g., probiotic agents, fish oil, inflammation, cyanocobalamin, dietary supplements), and Cluster III (blue) with 274 items (e.g., lysozyme, disease resistance, animal, growth, and upregulation). The network visualization of these keyword co-occurrences is shown in Figure 5.

## 13. Future Prospects and Conclusion

Although the effects of probiotics on growth, feed efficiency, gut microbiota, disease susceptibility, innate immune parameters, mucosal barriers, and cell damage/morphology of most aquatic animals have been thoroughly investigated, their detailed mechanisms of action remain largely unknown. Additionally, the specificity of probiotics concerning species, strain, and developmental stage is a crucial issue that needs to be addressed comprehensively and promptly. The future of probiotics in aquaculture is poised to evolve with advancements in biotechnology and a deeper understanding of microbial interactions in aquatic environments. Personalized probiotic formulations tailored to specific species, life stages, and environmental conditions will likely become more prevalent, enhancing both feed efficiency and disease resistance. The integration of probiotics with other emerging practices, such as prebiotics and biofloc systems, promises to further boost aquaculture productivity while maintaining ecosystem balance. As the industry moves toward more sustainable practices, probiotics will continue to be at the forefront of innovation, offering natural, eco-friendly solutions for improved fish health and growth. Further, the bibliometric analysis of probiotics research revealed a significant growth in studies focusing on their application in aquaculture, highlighting their potential as a sustainable solution. In conclusion, probiotics are set to play a pivotal role in driving aquaculture production, providing sustainable and eco-friendly solutions to enhance fish health, growth, and overall productivity. Leveraging insights from bibliometric analyses and fostering interdisciplinary collaboration, coupled with advancements in biotechnology, will be essential to unlocking their full potential and establishing more resilient and environmentally balanced aquaculture systems worldwide.

## Figures and Tables

**Table 1:** Application of probiotics in aquaculture.

Probiotic strain	Fish species	Dose	Results	Reference
*Bacillus subtilis* *Bacillus licheniformis*	Japanese eel	1 × 10^8^ CFU/g bacterial cells	Enhanced growth performance	Park et al. [13]
*Micrococcus luteus*	*Oreochromis niloticus*	10^10^ cells/kg diet	↑ WG, and ↓ FCR	Abd El-Rhman et al. [12]
*Bacillus thuringiensis* *B. megaterium* *B. polymyxa* *B. licheniformis* *B. subtilis*	(Post larvae) White shrimp	10^9^ CFU/ml daily 3–4 nauplii per shrimp 4 times daily	Effectively improves GR, survival, beneficial bacteria, water quality parameters	Nimrat et al. [28]
*L. acidophilus*	Common carp (*Cyprinus carpio*)	10^2^, 10^4^, or 10^6^ CFU/Kg diet	↑WG, SGR, FI and ↓FCR	Adeshina et al. [29]
*Bacillus* PC465	*Litopenaeus vannamei*	10^7^ CFU/g 10% of body weight 3 times daily for 30 days	↑ Biomass of microbes ↑ enzymes such as amylase, protease, and lipase, in the mid-gut	Chai et al. [30]
Dead *B. subtilis*, *L. acidophilus*, *Clostridium butyricum*, and *Saccharomyces cerevisiae*	Japanese flounder (*Paralichthys olivaceus*)	*B. subtilis* (>1.6 × 10^7^ CFU /g), *L. acidophilus* (>1.2 × 10^8^ CFU/g), *C. butyricum* (>2.0 × 10^7^ CFU/g) and *S. cerevisiae* (>1.6 × 10^7^ CFU/g)	↑ Immune response and disease resistance	Taoka et al. [31]
*L. lactis*	*L. vannamei*	10^7^ or 10^8^ CFU/g	↑ growth performance, digestive enzyme activity, and disease resistance	Adel et al. [15]
*B. subtilis*	White shrimp	10^5^ and 10^8^ CFU/g	↑ GR, resistance to *V. harveyi* and upregulation of immune gene expression.	Zokaeifar et al. [32]
*Bacillus* sp. and *Lactobacillus* sp.	*L. vannamei*	5.749 ± 0.67 × 10^4^ CFU/g	Enhanced nutrient production	de Paiva Maia et al. [33]
*Enterococcus casseliflavus*	*C. carpio*	1 × 10^9^ CFU/g	Enhancing growth performance, immunity, and disease resistance	Adel et al. [15]
*Bacillus* strains mixture	Nile tilapia (*O. niloticus*)	0.1–0.2 g /Kg diet	↑ growth, immunity, stress responses, gut health and function, as well as the water quality	Elsabagh et al. [34]
*Bacillus* spp.	*Fenneropenaeus indicus*	10^6^ CFU/ml	Improvements in growth parameters, ↑ survival and improved water quality	Ziaei-Nejad et al. [35]

Abbreviations: CFU: colony-forming units; FCR: feed conversion ratio; FI: feed intake; GR: growth rate; SGR: specific growth rate; WG: weight gain.

**Table 2:** Top 10 authors and countries publishing on probiotics in the field of aquaculture.

Authors	No. of publications	Country/Territory	No. of publications
Ringø, E.	54	China	948
Dawood, M. A. O.	50	India	578
Hoseinifar, S. H.	49	United States	506
Carnevali, O.	41	Iran	294
Van Doan, H.	35	Spain	287
Austin, B.	33	United Kingdom	237
Gram, L.	32	Indonesia	231
Zhou, Z.	30	Egypt	210
Balebona, M. C.	29	Brazil	202
Sun, Y. Z.	29	Australia	166

## Data Availability

The data that support the findings of this study are available from the corresponding author upon reasonable request.
